# Molecular determinants of skeletal muscle force loss in response to 5 days of dry immersion in human

**DOI:** 10.1002/jcsm.13559

**Published:** 2024-10-25

**Authors:** Mathias Velarde, Michel‐Yves Sempore, Valentine Allibert, Valérie Montel, Josiane Castells, Loïc Treffel, Angèle Chopard, Thomas Brioche, Laetitia Cochon, Jérome Morel, Bruno Bastide, Anne‐Cécile Durieux, Laurence Stevens, Damien Freyssenet

**Affiliations:** ^1^ Laboratoire Interuniversitaire de Biologie de la Motricité Université Jean Monnet‐Saint‐Etienne Saint Etienne France; ^2^ Département d'Anesthésie et Réanimation Centre Hospitalier Universitaire de Saint Etienne Saint Etienne France; ^3^ Univ. Lille, Univ. Artois, Univ. Littoral Côte d'Opale, ULR 7369 ‐ URePSSS ‐ Unité de Recherche Pluridisciplinaire Sport Santé Société Lille France; ^4^ Institut Toulousain d'Ostéopathie, IRF'O Toulouse France; ^5^ Australian Research Centre in Complementary and Integrative Medicine (ARCCIM), School of Public Health University of Technology Sydney Ultimo Australia; ^6^ DMEM, INRAE Université Montpellier Montpellier France

**Keywords:** Excitation‐contraction coupling, Microgravity, Muscle atrophy, Muscle disuse, Slow and fast isoforms of myofibrillar proteins

## Abstract

**Background:**

Astronauts in Earth's orbit experience microgravity, resulting in a decline of skeletal muscle mass and function. On Earth, models simulating microgravity have shown that the extent of the loss in muscle force is greater than the loss in muscle mass. The reasons behind this disproportionate loss of muscle force are still poorly understood. In the present study, we hypothesize that alongside the loss in skeletal muscle mass, modifications in the expression profile of genes encoding critical determinants of resting membrane potential, excitation‐contraction coupling and Ca^2+^ handling contribute to the decline in skeletal muscle force.

**Methods:**

Healthy male volunteers (*n* = 18) participated in a 5‐day dry immersion (DI) study, an Earth‐based model of simulated microgravity. Muscle force measurement and MRI analysis of the cross‐sectional area of thigh muscles were performed before and after DI. Biopsies of the *vastus lateralis* skeletal muscle performed before and after DI were used for the determination Ca^2+^ properties of isolated muscle fibres, molecular and biochemical analyses.

**Results:**

The extent of the decline in force, measured as maximal voluntary contraction of knee extensors (−11.1%, *P* < 0.01) was higher than the decline in muscle mass (−2.5%, *P* < 0.01). The decline in muscle mass was molecularly supported by a significant repression of the anabolic IGF‐1/Akt/mTOR pathway (−19.9% and −40.9% in 4E‐BP1 and RPS6 phosphorylation, respectively), a transcriptional downregulation of the autophagy‐lysosome pathway and a downregulation in the mRNA levels of myofibrillar protein slow isoforms. At the single fibre level, biochemical and tension‐pCa curve analyses showed that the loss in force was independent of fibre type (−11% and −12.3% in slow and fast fibres, respectively) and Ca^2+^ activation properties. Finally, we showed a significant remodelling in the expression of critical players of resting membrane potential (aquaporin 4: −24.9%, ATP1A2: +50.4%), excitation‐contraction coupling (*CHRNA1*: +75.1%, *CACNA2D1*: −23.5%, *JPH2*: −24.2%, *TRDN*: −15.6%, *S100A1*: +27.2%), and Ca^2+^ handling (*ATP2A2*: −32.5%, *CASQ1*: −15%, *ORAI1*: −36.2%, *ATP2B1*: −19.1%).

**Conclusions:**

These findings provide evidence that a deregulation in the expression profile of critical molecular determinants of resting membrane potential, excitation‐contraction coupling, and Ca^2+^ handling could be involved in the loss of muscle force induced by DI. They also provide the paradigm for the understanding of muscle force loss during prolonged bed rest periods as those encountered in intensive care unit.

## Introduction

Space flight has a significant effect on the health of astronauts. The unique environment of space with its lack of gravity, confinement, and exposure to radiation has a detrimental impact on the human body. This includes effects on cardiovascular and nervous systems, as well as musculoskeletal system.^1^, ^2^ The effect on skeletal muscle is notably characterized by a loss of mass and strength. Together, these adaptations to microgravity impede not only the ability of astronauts to perform tasks during spaceflight but also their post‐flight recovery capacity.^1^

The loss of muscle strength ranges from 10% to 40% depending on the duration of the spaceflight.^1^ On Earth, reductions in muscle strength have also been observed with models simulating microgravity. Decreases in knee extension strength have thus been reported after 10 days of bed rest (−14%)^3^ and 3 days of dry immersion (DI; −9%),^4^ a more severe model of simulated microgravity. Astronauts also experienced important reductions in muscle volume, ranging from 5% to 15% depending on the duration of the spaceflight.^1^ Ground‐based models simulating microgravity also trigger a loss of muscle mass, with reductions of 2% and 4% after 3 days of DI^4^ and 10 days of bed rest,^3^ respectively. Importantly, studies on Earth have consistently shown greater decline in strength (reaching up to −30% after a 3‐month bed rest) than in muscle mass (reaching up to −15% after a 3‐month bed rest).^5^

This disproportional loss of muscle force could be potentially attributed to all events involved in force production, from cortical activation to skeletal muscle contractions. A reduction in the conduction velocity of the motor neuron axon terminals and the muscle fibres,^6^ as well as a molecular remodelling of the neuromuscular junction,^3^, ^4^ could contribute to lower skeletal muscle strength in response to simulated microgravity. Changes in the composition of the extracellular matrix of skeletal muscle, previously described after DI,^7^ may also alter the contractile properties of muscle fibres. Additionally, alterations in the expression of slow and fast isoforms of myofibrillar proteins, which have been previously reported after unloading in humans,^8^, ^9^ may also contribute to alter the contractile properties of muscle fibres by modulating their shortening velocity and dependence on Ca^2+^.

There is currently no in‐depth analysis of the molecular determinants of skeletal muscle force in human skeletal muscle following simulated microgravity. We hypothesized that the expression of critical players of resting membrane potential, excitation‐contraction coupling (ECC) and Ca^2+^ handling is modified in response to 5 days of DI in human skeletal muscle. Furthermore, we aimed to determine whether a thigh cuff countermeasure, which has been shown to effectively alleviate cephalad fluid shift symptoms in the early stages of space travel,^10^ can provide benefits for skeletal muscle.

## Materials and methods

### Ethical approval

This study, conducted at the MEDES (Toulouse, France), followed the principles of the Declaration of Helsinki and was approved by the local ethic committee (RCB 2018‐A01470‐55) and French Health Authorities. The study was registered at ClinicalTrials.gov (NCT03915457).

### Participants

Eighteen healthy volunteers (age: 34.0 ± 5.5 years; height: 178 ± 6 cm; body mass: 74.1 ± 8.0 kg; body mass index: 23.3 ± 1.8 kg/m^2^) provided written informed consent. They had no history or physical sign of neuromuscular disorders, were non‐smokers, and did not take drugs or medication.

### Experimental protocol

**Figure 1:**
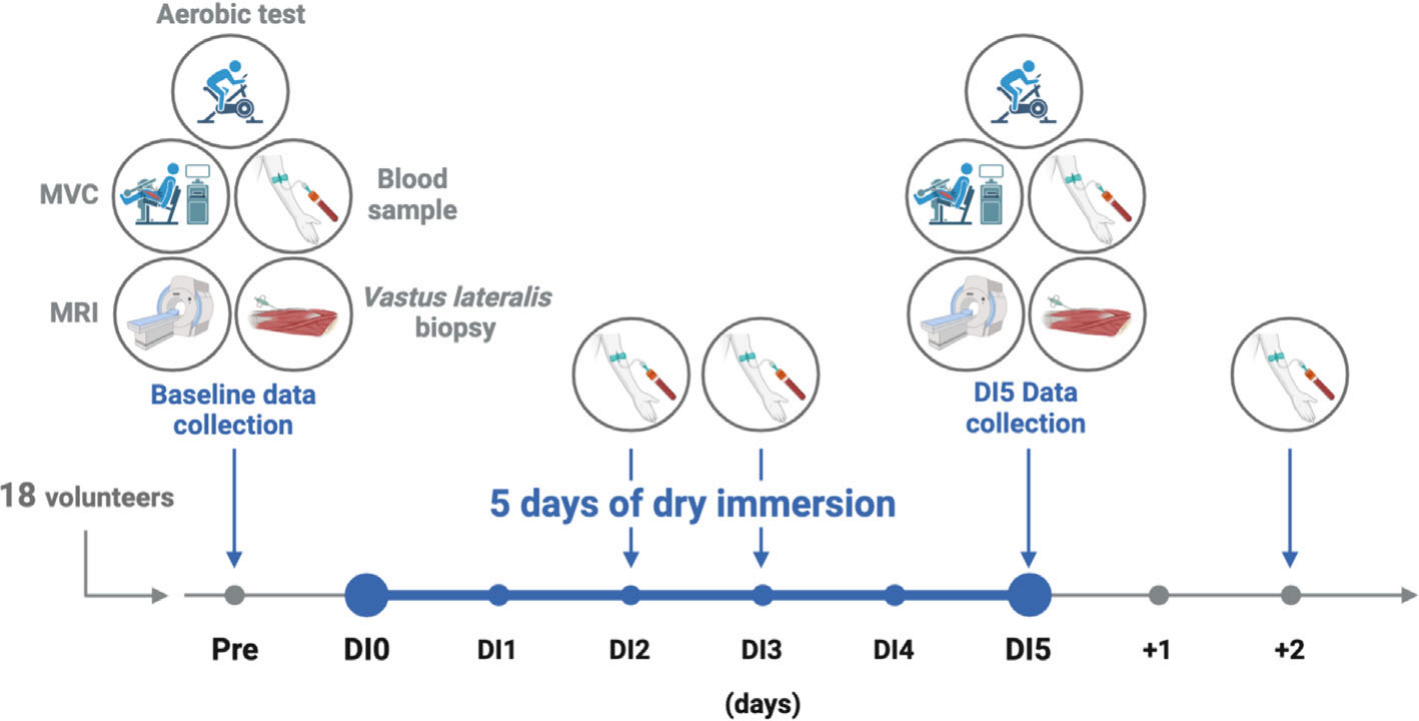
Schematic view of the dry immersion (DI) study. MRI, magnetic resonance imaging; MVC, maximal voluntary contraction; Pre, baseline data collection before DI.

Two participants simultaneously underwent DI in a supine position in two separate baths at thermoneutral water temperature (33 ± 0.5°C) in the same room with a light‐off period set at 23:00–07:00. Body weight, blood pressure, heart rate, tympanic body temperature, and dietary intake were individually controlled.^11^ During out‐of‐bath periods required for daily hygiene and specific measurements (9.7 ± 1.3 h over the duration of the study), the subjects were maintained in a −6° head‐down position. Data were acquired before DI (Pre), during DI and after 2 days of recovery (Figure 1).

### Thigh cuff countermeasure

Participants were randomly divided into a Control group and a Cuff group. Baseline characteristics were not different between Control and Cuff groups. Thigh cuffs were placed immediately prior to the onset of immersion at 10 pm. Participants wore thigh cuffs at 30–50 mmHg from 10 am to 6 pm on the first day of DI and from 8 am to 6 pm thereafter. Individual adjustments were made for each participant based on calf plethysmography measurements obtained with the participant in the supine position on the second day of DI.

### Maximal oxygen consumption

Oxygen uptake and heart rate were continuously monitored during an incremental test performed on a bicycle ergometer (Cardiowise) before and after DI. Volunteers cycled for 3 min at 50, 100, and 150 W, then followed by an increase of 25 W every 1 min until exhaustion.

### Magnetic resonance imaging

MRI acquisitions (Siemens‐Avanto 1.5 T, CHU Rangueil) were performed at Pre and at DI5. The placement of the magnetic field (FoV: 240 mm; repetition time: 6.98 ms; echo time: 2.38 ms; section thickness: 5 mm; 560 Hz/Px) was performed midway between the anterior‐superior iliac spine and the upper edge of the patella of the non‐biopsied left leg. The cross‐sectional area (CSA) of thigh muscles, defined as the sum of the CSA of the anterior and posterior compartments, was blindly determined by the same person using HOROS™.

### Maximal voluntary contraction

The maximal voluntary isometric contraction (MVC) strength of the knee extensors was measured on the non‐biopsied left leg at Pre and DI5 after familiarization using a ConTrex (Physiomed, Germany). Each measurement consisted of an extension movement followed by an isometric contraction at 80° extension maintained during 5–7 s. A set of three contractions with a 30‐s recovery period was recorded. MVC was defined as the maximum torque (Nm) achieved during the test.

### Blood sampling and analysis

Fasting blood samples were collected (7.30 am) in Vacutainer® EDTA K3 tubes at Pre, DI2, DI3, DI5, and after 48 h of recovery. Plasma samples were immediately frozen at −80°C after centrifugation. Myostatin and activin A concentrations were determined on undiluted serum according to the manufacturer's instructions (DGDF80 and DAC00B, R&D Systems).

### Skeletal muscle biopsy

A biopsy was performed on the right vastus lateralis muscle before DI and just before re‐ambulation on the last day of DI.^12^ The biopsies were obtained from areas as close as possible of the same vastus lateralis muscle. For mRNA and protein analyses, the tissue was rapidly frozen in liquid nitrogen and stored at −80°C. For tension‐pCa analysis, bundles of muscle fibres were oriented along the longitudinal axis, skinned, and finally stored at −20°C in a 50:50 glycerol‐skinning solution.^8^

### RNA isolation and reverse transcription‐quantitative polymerase chain reaction analysis

Total RNA was extracted (NucleoSpin RNA Set for NucleoZOL, Macherey‐Nagel). RNA (200 ng) was reverse transcribed (iScript cDNA synthesis, Bio‐Rad). Real‐time PCR was carried out on 2 μL of cDNA in a 10 μL final volume (Takyon No Rox SYBR MasterMix dTTP Blue, Eurogentec). Fluorescence intensity was recorded using a CFX96 Real‐Time PCR detection System (Bio‐Rad). Data were analysed using the ∆∆Ct method. Reference genes (18S RNA and beta‐2‐microglobulin) were used for normalization. Selected forward and reverse primers are listed in Table S1.

### Protein extraction and immunoblotting

Skeletal muscle was homogenized in a RIPA buffer (Cell Signaling Technology; 1:20 w:v) supplemented with protease and phosphatase inhibitors (Roche). After centrifugation (12 000 *g* at 4°C × 10 min), the supernatant was removed and kept at 4°C. The pellet was then resuspended in a RIPA buffer (1:10 w:v), frozen/thawed three times and centrifuged (12 000 *g* at 4°C × 10 min). Both supernatants were then combined for the measurement of protein concentration (DC Protein assay, Bio‐Rad). Twenty micrograms of proteins were electrophoresed on 4–20% pre‐cast gels (Bio‐Rad). For troponin C, T, and I, 30 μg of proteins were run on 10–20% gradient gels. Proteins were then transferred onto nitrocellulose membranes (Trans‐Blot Turbo Transfer System, Bio‐Rad). Membranes were blocked (TBS‐0.1% Tween 20 solution containing 5% non‐fat dried milk) for 2 h at room temperature and incubated overnight at 4°C with primary antibody (Table S2). Immune complexes were then probed with an appropriate secondary antibody (Table S2) for 2 h at room temperature. Membranes were imaged for fluorescence or chemiluminescence (ChemiDoc MP Imaging System, Bio‐Rad). Protein band intensity was determined (Image Lab 6.0, Bio‐Rad). Stain‐free technology (Bio‐Rad) was used for protein normalization of 4E‐BP1^Thr37/46^, AQP4, ATP1A2, NCX, TRPC1, RPS6, and RPS6^ser235/236^. For TnC, TnI, and TnT immunoblots, the optical density of band(s) corresponding either to slow or fast isoform(s) was expressed as the percentage of the sum of the optical densities of all the bands. This was done for each subject. Means therefore represent the relative distribution of the corresponding protein isoform.

### Tension‐pCa curve analysis

A 2–2.5 mm single fibre segment was isolated from the skinned biopsy and mounted in the experimental chamber.^13^ The fibres were then bathed in 2% Brij solution for 20 min.^14^ Diameter was measured and the fibre was stretched to 120% of its resting length to allow maximal tension development (P_0_).^13^ Sr^2+^ sensitivity of muscle fibres was used to identify 10 slow and 10 fast muscle fibres per subject.^8^ The tension‐pCa curve was then established by varying Ca^2+^ concentrations (pCa). Tension (P) was expressed as a fraction of P_0_ (P/P_0_).^13^ The tension‐pCa was fitted to the Hill equation.^15^

### Sodium dodecyl sulfate polyachrylamide gel electrophoresis analysis of myosin heavy chain and myosin light chain isoforms

Myofibrillar proteins were extracted^16^ from 40 mg of powdered muscle. Protein concentration was determined with a Lowry protein assay (Bio‐Rad). MHC and MLC isoforms were separated on 7.5% (1 μg protein/lane) and 12% gels (20 μg protein/lane), respectively. Gels were silver stained.^17^, ^18^ The signal intensities of the different protein isoforms were evaluated (Image Lab6.0, Bio‐Rad) and expressed as a percent of the sum of the signals of all isoforms.

### 2 dimension‐electrophoresis analysis of myosin light chain 2 phosphorylation

Proteins were separated by two‐dimensional gel electrophoresis.^18^ Proteins were first solubilized and then separated with the Ettan IPGphor Isoelectric Focusing System on 3.5% acrylamide strips with immobilized pH gradients^4^, ^5^, ^6^, ^7^ (Amersham Biosciences). After rehydration and electrofocusing, strips were embedded in 4% polyacrylamide stacking gels and the proteins were separated in 12% polyacrylamide mini gels (Bio‐Rad). Gels were then silver stained. Non‐phosphorylated and phosphorylated signals of slow and fast isoforms of MLC2 were analysed (Image Lab6.0, Bio‐Rad) and expressed as a percent of the sum of the total signal.

### Statistical analyses

Data are presented as means ± SD. Statistical analyses were performed using GraphPad Prism 9.2. Grubb's test was performed to identify outliers. Normal distribution was determined using the Shapiro–Wilk test. Two‐tailed paired *t*‐test or two‐tailed Wilcoxon test, one‐way ANOVA with repeated measures followed by Holm–Sidak multiple comparisons or Friedman test followed by Dunn's multiple comparisons, mixed‐effects model and Pearson correlation analysis were used. A description of the statistical analysis used for each variable is detailed in the figure legends. To assess the potential effect of the thigh cuff countermeasure, a principal component analysis has been performed. As we did not show any significant differences between Control and Cuff groups, data were presented as Pre‐ and DI5‐values. For information purposes, data from the Control and Cuff subjects were presented as empty and full circles, respectively. Statistical significance was considered when *P* < 0.05.

## Results

### Five days of dry immersion induce a greater loss in muscle strength than in muscle mass

**Figure 2:**
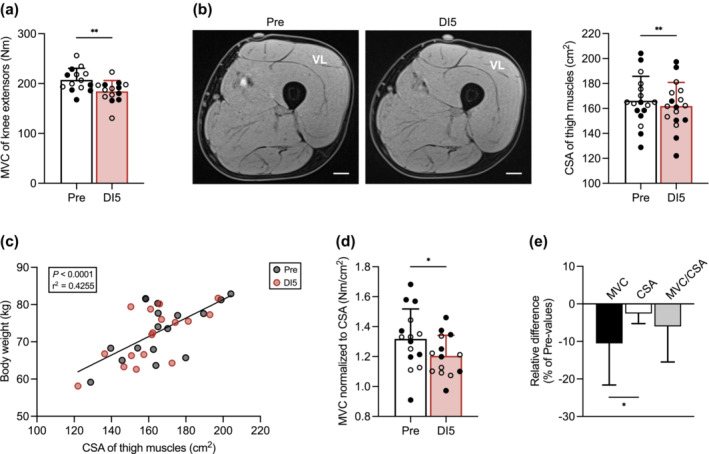
Five days of dry immersion (DI) induce a greater loss in muscle strength than in muscle mass. (A) Maximal voluntary contraction (MVC) of knee extensors (*n* = 14) at Pre and DI5. (B) Representative images (left) of thigh cross‐sectional area (CSA). Scale bars: 2 cm; VL (*vastus lateralis*); quantitative analysis (right) of the CSA of thigh muscles (*n* = 17). (C) Pearson correlation analysis between the CSA of thigh muscles and body weight (*n* = 17). (D) MVC normalized to thigh muscle CSA (*n* = 14). (E) Relative differences between DI5‐values and Pre‐values of MVC (*n* = 14), thigh muscle CSA (*n* = 17), and normalized MVC (*n* = 14). Data are means ± SD. Empty circles: Control subjects. Full circles: thigh cuff subjects. (A, B, D) Data were analysed by a paired *t*‐test. (E) Data were analysed by a one‐way ANOVA with Tukey's multiple comparisons test. *P < 0.05 and **P < 0.01.

DI induced significant reductions in body weight (−2.3%), VO_2max_ (−8.6%), and maximal aerobic power (−9.4%) (Table S3), together with an increase in maximal heart rate (Table S3). MVC of the knee extensors was significantly reduced (−11.1%) (Figure 2A),^19^ as well as the CSA of the thigh muscles (−2.5%) (Figure 2B). Additionally, thigh muscle CSA was significantly correlated with body weight (Figure 2C), indicating that the decline in skeletal muscle mass contributed to the loss in body weight. When normalized to the thigh muscle CSA, muscle force was still significantly lowered by DI (Figure 2D). Consequently, the relative loss in MVC exceeded that of thigh muscle CSA (Figure 2E). Collectively, these findings indicate that the decrease in muscle force cannot be solely explained by a reduction in muscle mass.

### Regulation of skeletal muscle proteostasis in response to 5 days of dry immersion

**Figure 3:**
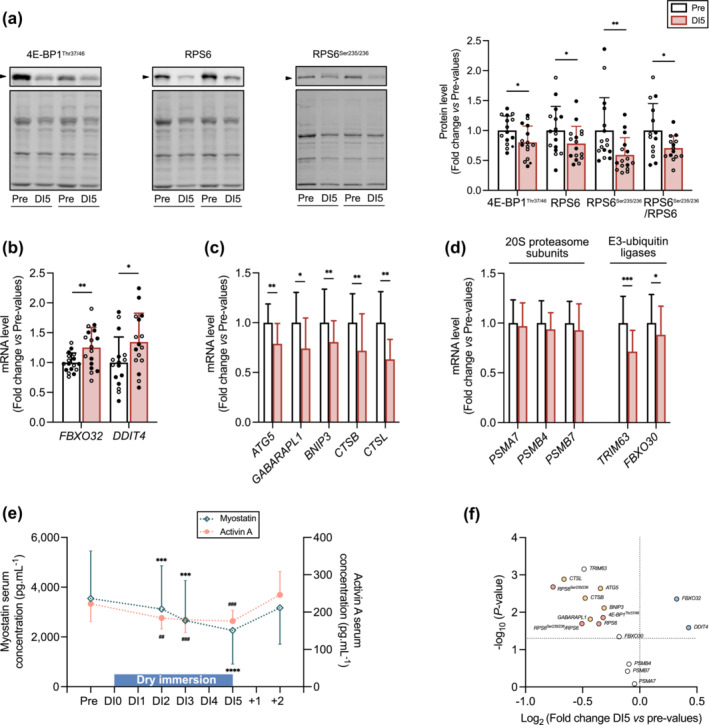
Regulation of skeletal muscle proteostasis in response to 5 days of dry immersion (DI). (A) Representative immunoblots (left) of 4E‐BP1^Thr37/46^, RPS6 and RPS6^Ser235/236^ protein content and corresponding stain free blots. Quantitative analysis (right) of 4E‐BP1^Thr37/46^ (*n* = 15), RPS6 (*n* = 16), RPS6^Ser235/236^ (*n* = 16) and RPS6^Ser235/236^/RPS6 (*n* = 14) protein content at Pre and DI5. (B) mRNA levels of *FBXO32* (*n* = 17) and *DDIT4* (*n* = 17). (C) mRNA levels of *ATG5* (*n* = 17), *GABARAPL1* (*n* = 17), *BNIP3* (*n* = 16), *CTSB* (*n* = 16), and *CTSL* (*n* = 17). (D) mRNA levels of *PSMA7* (*n* = 16), *PSMB4* (*n* = 16), *PSMB7* (*n* = 16), *TRIM63* (*n* = 17), and *FBXO30* (*n* = 15). (E) Myostatin and activin a serum concentrations at Pre, DI2, DI3, DI5, and 2 days after the cessation of DI (*n* = 18). (F) Volcano plot analysis of the molecular determinants of IGF‐1/Akt/mTOR pathway (pink), translational repressors (blue), autophagy‐lysosome pathway (yellow), 20S proteasome subunits and E3‐ubiquitin ligases (white). Data are means ± SD. Empty circles: Control subjects. Full circles: thigh cuff subjects. (A, B, C, D) Data were analysed by a two tailed paired *t*‐test except for RPS6^Ser235/236^ protein content, *CTSB* and *PSMA7* mRNA level that were analysed by a two tailed Wilcoxon test. *P < 0.05, **P < 0.01, and ***P < 0.001. (E) Myostatin serum concentration was analysed by a one‐way ANOVA with Holm–Sidak multiple comparisons. Activin A serum concentration was analysed by a Friedman test with Dunn's multiple comparisons. Significantly different from Pre‐values: ##*P* < 0.01, *** and ###*P* < 0.001, ****P < 0.0001.

DI resulted in the downregulation of the anabolic IGF‐1/Akt/mTOR pathway, as evidenced by the significant reductions in the phosphorylated inactive form of the translational repressor 4E‐BP1 and the active phosphorylated form and total protein content of RPS6 (Figure 3A). RPS6 total protein content was also significantly decreased, as well as the ratio of the phosphorylated‐to‐total forms of RPS6. This was also associated with an increase in the mRNA level of the translational repressors *FBXO32* and *DDIT4* (Figure 3B).

The transcript levels of key molecular determinants of the autophagy‐lysosome proteolytic pathway, including *ATG5*, *GABARAPL1*, *BNIP3*, *CTSB*, and *CTSL*, were significantly reduced after DI (Figure 3C). Whereas expression of *PSMA7*, *PSMB4*, and *PSMB7*, encoding 20S proteasome subunits, remained unchanged (Figure 3D), the transcript levels of *TRIM63* and *FBXO30*, which encode E3 ligases of the ubiquitin‐proteasome proteolytic pathway (Figure 3D), as well as the serum concentration of the extracellular catabolic factors myostatin and activin A (Figure 3E), were also lowered in response to DI. All the data are summarized in Figure 3F.

### Five days of dry immersion downregulate the expression of myofibrillar proteins and induce a shift towards a faster phenotype

**Figure 4:**
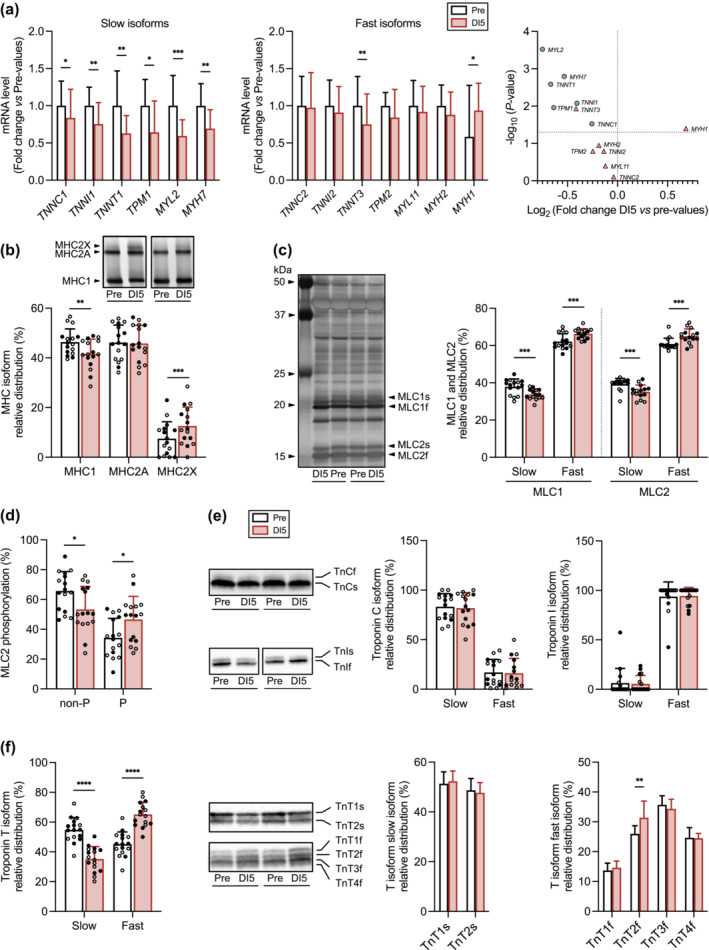
Effects of 5 days of dry immersion (DI) on the expression of myofibrillar proteins. (A) Transcript levels of the slow isoforms (left) of troponin C (*TNNC1*; *n* = 17), troponin I (*TNNI1*; *n* = 17), troponin T (*TNNT1*; *n* = 17), tropomyosin (*TPM1*; *n* = 16), MLC (*MYL2*; *n* = 16), and MHC (*MYH7*; *n* = 16); transcript levels of the fast isoforms (center) of troponin C (*TNNC2*; *n* = 17), troponin I (*TNNI2*; *n* = 17), troponin T (*TNNT3*; *n* = 17), tropomyosin (*TPM2*; *n* = 17), MLC (*MYL11*; *n* = 16), MHC2a (*MYH2*; *n* = 15), and MHC2x (*MYH1*; *n* = 13); (left) volcano plot showing the fold change in myofibrillar protein mRNA level encoding slow (blue) and fast (pink) isoforms. (B) Representative SDS‐PAGE (upper) and quantitative analysis (lower) of the relative distribution of MHC1, MHC2A, and MHC2X at Pre and DI5 (*n* = 16). (C) Representative SDS‐PAGE (left) and quantitative analysis (right) of the relative distribution of MLC1 and MLC2 slow and fast isoforms at Pre and DI5 (*n* = 15). (D) Relative distribution of non‐phosphorylated (non‐P) and phosphorylated (P) forms of MLC2 (*n* = 16). (E) Representative immunoblot (left) and quantitative analysis (right) of the relative distribution of troponin C and troponin I slow and fast isoforms at Pre and DI5 (*n* = 14). (F) Quantitative analysis (left) of the relative distribution of slow and fast troponin T isoforms (*n* = 16). Representative immunoblots (middle left). Quantitative analysis (middle right) of the relative distribution of slow troponin T isoforms (*n* = 16). Quantitative analysis (right) of the relative distribution of fast troponin T isoforms (*n* = 16). Data are means ± SD. Empty circles: control subjects. Full circles: thigh cuff subjects. (A–F) Data were analysed by a two tailed paired *t*‐test except for *TPM1*, *MYH7*, and *MYH1* mRNA levels, MLC1, MLC2, TnCf, TnIs, TnIf, and TnT1f protein content that were analysed by a Wilcoxon test. *P < 0.05, **P < 0.01, ***P < 0.001 and ****P < 0.0001.

Transcript levels encoding the slow isoforms of MHC, MLC, troponin C, troponin I, troponin T, and tropomyosin were all reduced by DI, whereas those encoding for the fast isoforms remained unaffected or even increased (*MYH1*) (Figure 4A). SDS‐PAGE analysis of the relative distribution of MHC and MLC slow and fast isoforms indicated a shift towards a faster phenotype (Figure 4B,C). The phosphorylated forms of MLC2, previously associated with a slow‐to‐fast transition,^18^ were increased at the expense of the non‐phosphorylated ones (Figure 4D). Although the relative distribution of troponin C and troponin I slow and fast isoforms remained unchanged (Figure 4E), the relative distribution of the fast isoform of troponin T (TnTf) increased, while that of the slow isoform (TnTs) decreased (Figure 4F). Finally, the relative distribution within the TnTs isoforms (TnT1s and TnT2s) remained unchanged, while that of TnT2f (one of the four TnTf isoforms) was increased.

### Five days of dry immersion lower Ca^2+^‐induced maximal tension of slow and fast muscle fibres and alters the tension‐pCa curve of slow muscle fibres

**Figure 5:**
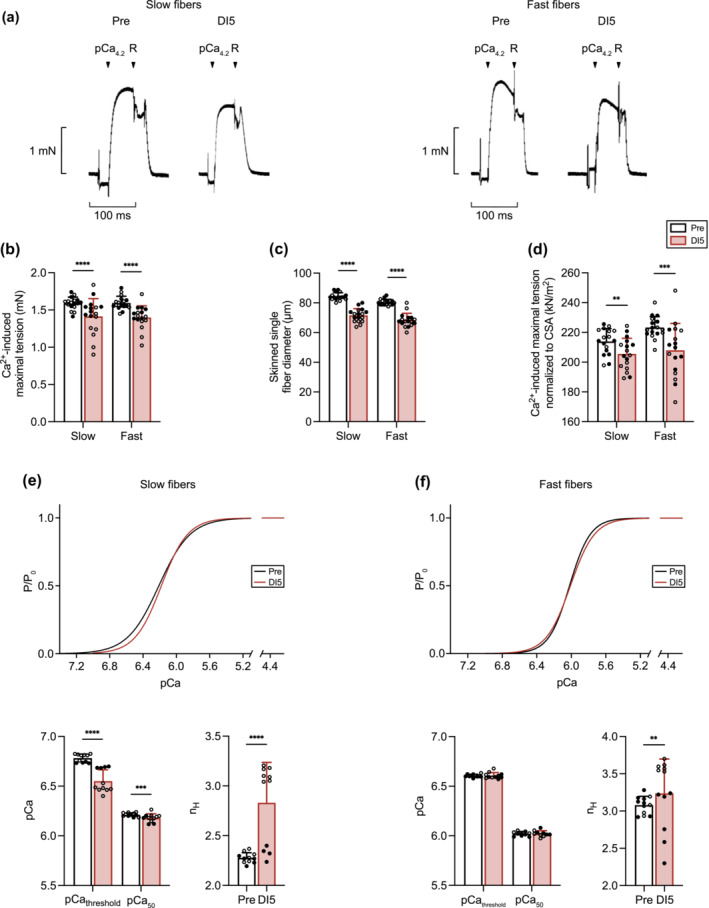
Five days of dry immersion (DI) lower Ca^2+^‐induced maximal tension of slow and fast muscle fibres and alters the tension‐pCa curve of slow muscle fibres. (A) Representative recordings used to determine the Ca^2+^‐induced maximal tension of a slow (left) and fast (right) fibre before and after DI. Ca^2+^‐induced maximal tension was induced by a pCa_4.2_ solution. Relaxation was obtained by using a relaxing solution (R) that does not contain Ca^2+^. (B) Ca^2+^‐induced maximal tension (*n* = 17). (C) Skinned muscle fibre diameter (*n* = 17). (D) Ca^2+^‐induced maximal tension normalized to fibre cross‐sectional area (CSA) of slow and fast muscle fibres (*n* = 17). (E) Mean tension‐pCa curves (upper) of slow fibres at Pre and DI5 obtained by Hill equation. (lower left) Ca^2+^ lowest concentration required to generate a tension (pCa_threshold_) and Ca^2+^ concentration required to generate 50% (pCa_50_) of the maximal tension (*n* = 11). (Lower right) Hill coefficient (n_H_) of slow fibres at Pre and DI5 (*n* = 11). (F) Mean tension‐pCa curves (upper) of fast fibres at Pre and DI5 obtained by Hill equation. (lower left) pCa_threshold_ and pCa_50_ (*n* = 10–11). (Lower right) n_H_ of fast fibres at Pre and DI5 (*n* = 12). A total of 10 slow and 10 fast fibres per subject was analysed. Each individual point represents the mean of 10 fibres. Data are means ± SD. Empty circles: control subjects. Full circles: thigh cuff subjects. All data were analysed by a mixed‐effects model. **P < 0.01, ***P < 0.001, and ****P < 0.0001.

In agreement with MVC data (Figure 2A,D), Ca^2+^‐induced maximal tension, diameter of skinned muscle fibres, and Ca^2+^‐induced maximal tension normalized to muscle fibre CSA (Figure 5B–D) were significantly reduced both in slow and fast fibres. The tension‐pCa curve showed that DI decreased pCa threshold and pCa_50_ in slow fibres, whereas Hill number (n_H_) was increased both in slow and fast fibres (Figure 5E,F).

### Five days of dry immersion induce changes in the expression of molecular determinants of resting membrane potential and excitation‐contraction coupling

**Figure 6:**
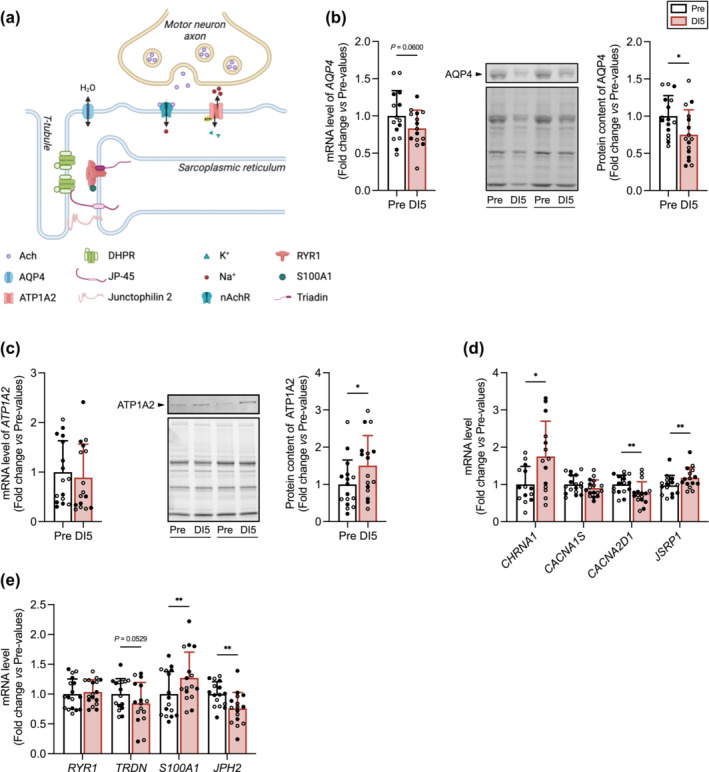
Five days of dry immersion (DI) induce changes in the expression of molecular determinants of resting membrane potential and excitation‐contraction coupling. (A) Schematic view of the molecular targets analysed. Aquaporin 4 (AQP4) regulates water movement across the sarcolemma. *ATP1A2* encodes the Na^+^/K^+^‐transporting ATPase subunit alpha‐2. *CHRNA1* encodes the alpha1 subunit of the nicotinic acetylcholine receptor (nAchR). *CACNA1S* encodes the voltage‐dependent L‐type Ca^2+^ channel subunit alpha‐1S of the dihydropyridine receptor (DHPR). *CACNA2D1* encodes the voltage‐dependent Ca^2+^ channel subunit alpha‐2/delta‐1 of the DHPR. *JSRP1* encodes the junctional sarcoplasmic reticulum protein 1 (JP‐45) involved in the regulation of the DHPR. RYR1 (ryanodine receptor 1) is a Ca^2+^ release channel of the sarcoplasmic reticulum that opens in response to depolarization. *TRDN* encodes triadin, a protein required for normal organization of the triad and the regulation of Ca^2+^ release through RYR1. S100A1 is a RYR1 regulatory protein. *JPH2* encodes junctophilin 2, a membrane‐binding protein that provides a structural bridge between the plasma membrane and the sarcoplasmic reticulum. (B) *AQP4* mRNA level (left) at Pre and after DI5 (*n* = 15). Representative immunoblot and corresponding stain free blot (middle) of AQP4. Quantitative analysis (right) of AQP4 protein content at Pre and DI5 (*n* = 16). (C) *ATP1A2* mRNA level (left) at Pre and DI5 (*n* = 16). Representative immunoblot and corresponding stain free blot (middle) of ATP1A2. Quantitative analysis (right) of ATP1A2 protein content at Pre and DI5 (*n* = 16). (D) mRNA levels of *CHRNA1* (*n* = 14), *CACNA1S* (*n* = 17), *CACNA2D1* (*n* = 16), and *JSRP1* (*n* = 15) at Pre and DI5. *(E)* mRNA levels of *RYR1* (*n* = 17), *TRDN* (*n* = 16), *S100A1* (*n* = 16), and *JPH2* (*n* = 16) at Pre and DI5. Data are means ± SD. Empty circles: control subjects. Full circles: thigh cuff subjects. Data were analysed by a two tailed paired *t*‐test except for *ATP1A2* and *S100A1* mRNA levels that were analysed by a Wilcoxon test. *P < 0.05 and **P < 0.01.

We next examined the expression of molecular determinants involved in establishing the resting membrane potential (Figure 6A), including AQP4, the main aquaporin isoform in skeletal muscle,^20^ and ATP1A2 (ATPase Na^+^/K^+^ transporting subunit alpha 2). Transcript and protein levels of AQP4 were reduced (Figure 6B, Table S4). Although *ATP1A2* mRNA level remained unchanged (Figure 6C, Table S4), the protein content was significantly increased (Figure 6C).

We next investigated the expression of ECC critical players (Figure 6A). The transcript level of *CHRNA1* (acetylcholine receptor alpha‐1 subunit) was significantly upregulated following DI (Figure 6D, Table S4). mRNA level of *CACNA1S* (Cav1.1) of the dihydropyridine receptor and *RYR1* (Figure 6D,E and Table S4) remained unchanged. Transcript levels of *CACNA2D1* and *JSRP1* encoding Cav1.1 regulatory proteins and *TRDN*, *S100A1*, and *JPH2* encoding RYR1 interacting proteins were all modified (Figure 6D,E, Table S4). Together, these data point to an altered expression profile of components of resting membrane potential and ECC in response to DI.

### Five days of dry immersion alter the expression of molecular determinants of Ca^2+^ handling

**Figure 7:**
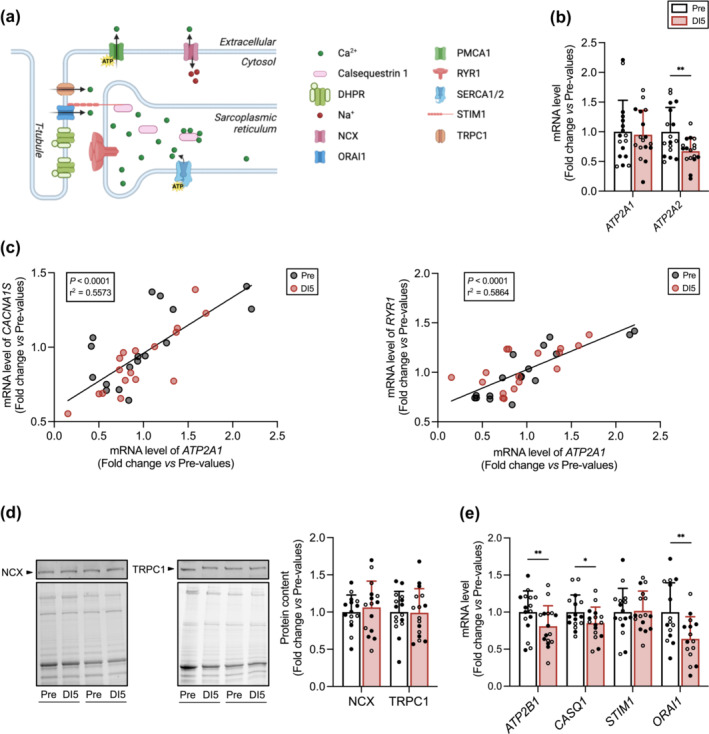
Five days of dry immersion (DI) alter the expression of molecular determinants of Ca^2+^ handling. (A) Schematic view of the molecular targets analysed. *ATP2A1* and *ATP2A2* encode the sarcoplasmic/endoplasmic reticulum Ca^2+^ ATPase fast (SERCA1) and slow (SERCA2) isoforms, respectively. *CASQ1* encode calsequestrin 1, a reticulum sarcoplasmic Ca^2+^ buffer protein. Upon sarcoplasmic reticulum Ca^2+^ store depletion, stromal interaction molecule 1 (STIM1) activates the Ca^2+^ release‐activated Ca^2+^ channel protein 1 (ORAI1). They both play a critical role in mediating store‐operated Ca^2+^ entry (SOCE), an extracellular Ca^2+^ influx following depletion of intracellular Ca^2+^ store. Short transient receptor potential channel 1 (TRPC1) is a non‐selective Na^+^/Ca^2+^ channel located on the sarcolemma. Na^+^/Ca^2+^ exchanger (NCX) is located on the sarcolemma and removes Ca^2+^ from the cytosol. *ATP2B1* encodes the plasma membrane Ca^2+^‐transporting ATPase 1 (PMCA1), which catalyses the hydrolysis of ATP coupled with the transport of Ca^2+^ from the cytoplasm to the extracellular space. (B) mRNA levels of *ATP2A1* and *ATP2A2* at Pre and DI5 (*n* = 17). (C) Pearson correlation analysis between *CACNA1S* and *ATP2A1* mRNA levels (left) and *RYR1* and *ATP2A1* mRNA levels (right). (D) Representative immunoblots and corresponding stain free blots (left) and quantitative analysis (right) of NCX (*n* = 15) and TRPC1 (*n* = 16) protein content at Pre and DI5. (E) mRNA levels of *ATP2B1* (*n* = 16), *CASQ1* (*n* = 16), *STIM1* (*n* = 16), and *ORAI1* (*n* = 15) at Pre and DI5. Data are means ± SD. Empty circles: control subjects. Full circles: thigh cuff subjects. (B, D, E) Data were analysed by a two tailed paired *t*‐test except for *ATP2A1* and *CASQ1* mRNA levels that were analysed by a Wilcoxon test. *P < 0.05 and **P < 0.01.

We finally determined the expression of molecular determinants of Ca^2+^ handling (Figure 7A). Although mRNA level of *ATP2A1* mRNA (SERCA1 fast isoform) remained unchanged, that of *ATP2A2* (SERCA2 slow isoform) was significantly downregulated after DI (Figure 7B, Table S4). Interestingly, *ATP2A1* mRNA level was correlated with *CACNA1S* and *RYR1* mRNA levels (Figure 7C), illustrating the functional relationship between the dihydropyridine receptor, RYR1 and SERCA1.

The protein levels of sarcolemmal Ca^2+^ transport regulators including, NCX and TRPC1, remained unchanged (Figure 7D), whereas mRNA level of *ATP2B1* (plasma membrane Ca^2+^ ATPase 1) decreased in response to DI (Figure 7E, Table S4). Finally, *CASQ1* mRNA level was also decreased (Figure 7E, Table S4). ORAI1 is essential in regulating Ca^2+^ entry into the cytosol in response to the depletion of SR Ca^2+^ store sensed by STIM1.^21^ While *STIM1* mRNA level remained unaffected after DI, *ORAI1* transcript level was downregulated (Figure 7E, Table S4). Therefore, DI induced a marked dysregulation in the expression of critical molecular determinants of Ca^2+^ handling.

### Thigh cuff countermeasure does not modify the molecular response induced by dry immersion

**Figure 8:**
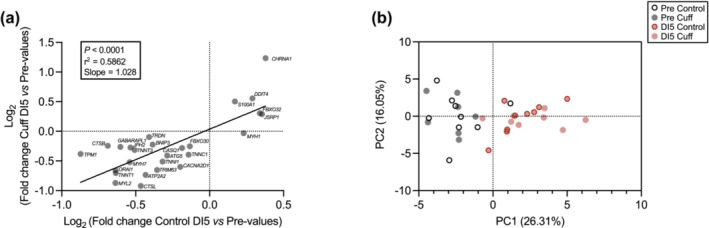
Thigh cuff countermeasure did not modify the molecular response induced by dry immersion (DI). (A) Pearson correlation analysis of Log_2_ fold change of all significantly regulated transcripts between Control and Cuff subjects. (B) Principal component analysis was performed by using all significantly regulated transcripts.

We finally ask whether a thigh cuff countermeasure can alter the transcriptional response induced by DI. The fold change in gene expression between Control and Cuff groups was strongly correlated (Figure 8A; *P* < 0.0001). Notably, the slope value (1.028; 0.6644 < 95% CI < 1.392) suggests similarities in the extent of the transcriptional response between Control and Cuff groups. A principal component analysis further showed that, whereas Pre and DI5 subjects could be separated, Control and Cuff subjects could not be discriminated after DI (Figure 8B). Collectively, these data indicate that the thigh cuff countermeasure did not modify the molecular response elicited by DI.

## Discussion

In the present study, the disproportionate loss of muscle force induced by DI, when compared with muscle mass loss, was associated with a shift towards a fast‐like profile. We further showed that a marked alteration in the expression profile of multiple molecular determinants of membrane resting potential, ECC, and Ca^2+^ handling could potentially contribute to explain this loss in muscle force.

The decrease in knee extensor MVC after 5 days of DI (−11.1%) was very similar to that previously reported after 3 days of DI (−10%).^4^ A similar decrease was also observed in response to a bed rest protocol, but after a longer period of immobilization (10 days),^3^ highlighting the greater severity of the DI model. The reduction in thigh muscle CSA (−2.5%), which agrees with previous DI^4^ and bed rest^3^ data, was much lower than the decline in muscle force, indicating that the decrease in muscle force could not entirely explain the decrease in muscle mass. This was also corroborated by our measurements of Ca^2+^‐induced maximal tension on isolated muscle fibres.

Skeletal muscle mass is finely tuned by several signalling pathways.^22^ The downregulation of the anabolic IGF‐1/Akt/mTOR pathway, together with the up‐regulated expression of translational repressors (*FBXO32* and *DDIT4*), agreed with previous data showing a decrease in basal protein synthesis rate in response to disuse atrophy in humans.^23^ The autophagy‐lysosome pathway undergoes strong regulation at the transcriptional level.^22^ Here, the lower expression of autophagy‐related genes in response to DI may indicate a decline in the autophagic process. Prior observations have shown that autophagy‐deficient mice displayed muscle atrophy.^24^ Our data suggest that such a scenario might compromise skeletal muscle homeostasis, consequently leading to muscle atrophy. Conversely, our data could alternatively signify an adaptation of skeletal muscle to the hypokinetic state induced by DI.

The diminished expression levels of *TRIM63* (MuRF1) and *FBXO30* (MUSA1) suggest a potential decrease in MuRF1‐ and MUSA1‐targeted protein degradation through the ubiquitin‐proteasome pathway. This observation aligns with the reduced levels of circulating catabolic factors myostatin and activin A. However, this cannot also exclude the possibility that the expression of E3 ligases and myostatin have increased earlier during the DI. Indeed, data from animal studies have convincingly demonstrated an early and timely regulated response of E3 ligases and myostatin during muscle disuse.^25^, ^26^ Finally, the reduction in the pool of translatable mRNA encoding the slow isoforms of myofibrillar proteins (MHC, MLC, troponin C, troponin I, troponin T, tropomyosin), without any compensatory increase in the mRNA level encoding fast isoforms, could also contribute to decrease muscle mass by lowering the capacity for myofibrillar protein synthesis. In summary, our analyses suggest that, notwithstanding the reduction in the expression of *TRIM63* and *FBXO30* and a lower pressure of extracellular catabolic factors (myostatin and activin A), an hypo‐anabolism, coupled with a downregulation of the autophagy‐lysosome pathway and a decrease in the pool of translatable mRNA encoding myofibrillar proteins, may contribute to the loss of skeletal muscle mass after 5 days of DI.

The reduction in the mRNA levels encoding the slow isoforms of myofibrillar proteins also indicates a transition towards a faster phenotype. This is further corroborated by the increase in the relative distribution of the fast protein isoforms of MHC, MLC, and troponin T, as well as by the increase in the phosphorylation of MLC2.^18^ Notably, the transition to faster isoforms was not detected for troponin C and troponin I, indicating the existence of varying temporal responses among myofibrillar proteins.^16^ This could be due to differences in protein half‐life and the existence of distinct mechanisms regulating expression of myofibrillar proteins. The data acquired at the single fibre level also provide additional evidence for a transition towards a faster phenotype. In comparison with slow muscle fibres, the tension‐pCa curve of fast fibres is characterized by a lower Ca^2+^ sensitivity and a higher cooperativity.^13^ Here and in line with previous findings,^8^, ^27^ the lower pCa threshold and higher n_H_ in slow muscle fibres after DI strongly suggests that the functional properties of slow muscle fibres more closely resemble those of fast fibres. This could be ascribed to a shift towards the expression of fast TnT isoforms,^28^ as supported by the increase in the relative distribution of TnT fast isoforms (TnT2f).

As previously described in other conditions of muscle inactivity in humans,^8^, ^29^ the reduction in Ca^2+^‐induced maximal tension in slow and fast fibres indicates that the loss in force was independent of fibre type. This loss of force did not seem to be due to changes in Ca^2+^ activation properties, given that both slow and fast fibres lost force, whereas slow fibres mainly displayed modified Ca^2+^ activation properties. MLC2 phosphorylation has been documented to increase myofibril Ca^2+^ affinity and force development.^18^ However, the increase in MLC2 phosphorylation after DI, also reported in other models of muscle disuse,^18^, ^30^ does not agree with this scenario and could rather be interpreted as an unsuccessful attempt to limit the extent of skeletal muscle force loss. Collectively, these data thus point to a shift towards a fast profile associated with a decrease in muscle force.

The decrease in muscle force, irrespective of muscle fibre type and Ca^2+^ activation properties of myofibrillar proteins, suggests significant DI‐related changes in the expression of critical molecular determinants of force production. Here, the altered expression of 13 out of 27 mRNA and proteins supports this hypothesis. The reduction in AQP4 expression following DI, also documented in other conditions of muscle inactivity,^31^ could be interpreted as an adaptation to a reduction in water movement across the sarcolemma, that would be consecutive to the reduction in hydrostatic pressure in lower limbs caused by the redistribution of blood towards the head. The changes in AQP4 and ATP1A2 (Na^+^/K^+^‐ATPase pump) protein levels after DI also question the functional relevance of these variations in setting the resting membrane potential and thus muscle fibre excitability. Interestingly, gene targeting of AQP4 in mice leads to premature fatigue,^32^ suggesting that the decrease in AQP4 protein level after DI may decrease fatigue resistance during prolonged contractions.

The upregulation of *CHRNA1* mRNA level agrees with previous studies showing increased expression of acetylcholine receptor subunits in denervated mouse muscle fibres^33^ or in response to muscle inactivity in human^3^, ^34^ and may indicate an altered neuro‐muscular connection or an attempt to counteract the decrease in neural drive.^6^ Notably, acetylcholine receptor and Na^+^/K^+^‐ATPase pump have been shown to colocalize at the postsynaptic membrane,^35^ suggesting that the upregulation of *CHRNA1* mRNA level and ATP1A2 protein content observed here may be part of a coordinated response.

Skeletal muscle triads house the ion channel machinery of ECC, Cav1.1 (*CACNA1S*), and RYR1, together with a variety of proteins involved in the maintenance of SR spatial organization and integrity (junctophilin 2), and the regulation of Cav1.1 (CACNA2D1, JP‐45) and RYR1 (triadin, calsequestrin 1, S100A1). Assuming that changes in mRNA levels are translated into changes in protein levels, the altered transcript levels of *JPH2* (junctophilin 2), *CACNA2D1*, *JSRP1* (JP‐45), *TRDN*, *S100A1*, and *CASQ1* should modify the stoichiometric ratios of functionally related proteins and thus potentially contribute to impair ECC and force production. Supporting this hypothesis, loss‐of‐function studies have shown altered Ca^2+^ dynamics in junctophilin 2 deficient myotubes^36^ and triadin knockout mice,^37^ together with a loss of muscle force. Additionally, changes in the expression of molecular determinants of ECC could also explain the decrease in Ca^2+^ release from the SR previously described in response to 10 days of bed rest.^3^

The reduction in *ATP2A2* mRNA level, encoding the slow SERCA isoform, agrees with the downregulation of slow myofibrillar protein isoforms mentioned earlier. The reduction in *ATP2A2*, *CASQ1*, and *ORAI1*, which encode key regulators of Ca^2+^ availability in the SR, may represent a transcriptional response to a reduction in sarcoplasmic Ca^2+^ content, a decrease previously described following unilateral limb suspension.^38^ Interestingly, dominant negative ORAI1 mice displayed decreased skeletal muscle force production and increased susceptibility to fatigue.^39^ Finally, the lower transcript level of *ATP2B1* (PMCA1) further provides molecular evidence of altered expression of Ca^2+^ handling proteins to DI.

In conclusion, the present investigation demonstrates that 5 days of DI elicits a more pronounced decrease in muscle force than in muscle mass. Our results provide the first experimental evidence that such a short duration of simulated microgravity is sufficient to alter the expression of the molecular determinants governing resting membrane potential, ECC and Ca^2+^ handling. These findings underscore that the close coordinated expression of molecular determinants of skeletal muscle force is lost in response to DI, potentially affecting Ca^2+^ dynamics during contractions, thus compromising force production and fatigue resistance. This is essential for the understanding of muscle force loss during microgravity, but also on Earth during extended periods of bed rest, such as observed in intensive care units.

## Conflict of interest

The authors declare that they have no conflict of interest.

## Supporting information


**Table S1.** Primer sequences used for qPCR analyses. *18S*: 18S ribosomal RNA; *AQP4*: aquaporin 4; *ATG5*: autophagy related 5; *ATP1A1* and *ATP1A2*: ATPase Na^+^/K^+^ transporting subunit alpha 1 and 2; *ATP2A1* and *2*: ATPase sarcoplasmic/endoplasmic reticulum Ca^2+^ transporting 1 and 2 (SERCA1 and SERCA2); *ATP2B1*: ATPase plasma membrane Ca^2+^ Transporting 1 (PMCA1); *B2M*: beta‐2‐microglobulin; *BNIP3*: BCL2 interacting protein 3; *CACNA1S*: Ca^2+^ voltage‐gated channel subunit alpha 1 S; *CACNA2D1*: Ca^2+^ voltage‐gated channel auxiliary subunit alpha 2 delta 1; *CALM1*: calmodulin 1; *CALU*: calumenin; *CASQ1*: calsequestrin 1; *CHRNA1*: cholinergic receptor nicotinic alpha 1 subunit; *CLCN1*: chloride voltage‐gated channel 1; *CTSB*: cathepsin B; *CTSL*: cathepsin L; *DDIT4*: DNA damage inducible transcript 4; *FBXO30*: F‐box protein 30; *FBXO32*: F‐box protein 32 (MAFBX); *GABARAPL1*: GABA type A receptor associated protein like 1; *JPH2*: junctophilin 2; *JSRP1*: junctional sarcoplasmic reticulum protein 1; *MYH1*, *MYH2* and *MYH7*: myosin heavy chain 1, 2 and 7 (MHC2A, MHC2X and MHC1, respectively); *MYL2* and *MYL11*: myosin light chain 2 and 11; *ORAI1*: ORAI Ca^2+^ release‐activated Ca^2+^ modulator 1; *PSMA7:* 20S proteasome subunit alpha 7; *PSMB4* and *PSMB7*: 20S proteasome subunit beta 4 and beta 7; *RYR1*: ryanodine receptor 1; *S100A1*: S100 Ca^2+^ binding protein A1; *SCN4A*: Na^+^ voltage‐gated channel alpha subunit 4; *SLC8A1*: solute carrier family 8 member A1; *SLN*: sarcolipin; *STIM1*: stromal interaction molecule 1; *TNNC1* and *TNNC2*: troponin C 1 and 2; *TNNI1* and *TNNI2*: Troponin I 1 and 2; *TNNT1* and *TNNT 3*: troponin T 1 and 3; *TPM1* and *TPM2*: tropomyosin 1 and 2; *TRDN*: triadin; *TRIM63*: tripartite motif containing 63 (MURF1).

**Table S2.** List of antibodies. 4E‐BP1^Thr37/46^: phosphorylated eIF4E‐binding protein 1; AQP4: aquaporin 4; ATP1A2: ATPase Na^+^/K^+^ transporting subunit alpha 2; NCX: Na^+^/Ca^2+^ exchanger; RPS6: Ribosomal protein S6; RPS6^Ser235/236^ phosphorylated ribosomal protein S6; TnCs: troponin C slow isoform; TnCf: troponin C fast isoform; TnIs: troponin I slow isoform; TnIf: troponin I fast isoform; TnTs: troponin T slow isoform; TnTf: troponin T fast isoform; TRPC1: transient receptor potential cation channel subfamily C member 1.

**Table S3** Effects of 5 days of DI on body weight, maximal heart rate, maximal oxygen consumption (VO_2_max) and maximal aerobic power. DI5: 5 days of dry immersion. Values are means ± SD and were analysed by two tailed paired *t*‐test.

**Table S4** Fold‐change in mRNA level. *AQP4*: aquaporin 4; *ATP1A1* and *ATP1A2*: ATPase Na^+^/K^+^ transporting subunit alpha 1 and 2; *ATP2A1* and *2*: ATPase sarcoplasmic/endoplasmic reticulum Ca^2+^ transporting 1 and 2 (SERCA1 and SERCA2); *ATP2B1*: ATPase plasma membrane Ca^2+^ Transporting 1 (PMCA1); *CACNA1S*: Ca^2+^ voltage‐gated channel subunit alpha 1 S; *CACNA2D1*: Ca^2+^ voltage‐gated channel auxiliary subunit alpha 2 delta 1; *CALM1*: calmodulin 1; *CALU*: calumenin; *CASQ1*: calsequestrin 1; *CHRNA1*: cholinergic receptor nicotinic alpha 1 subunit; *CLCN1*: chloride voltage‐gated channel 1; *JPH2*: junctophilin 2; *JSRP1*: junctional sarcoplasmic reticulum protein 1; *ORAI1*: ORAI Ca^2+^ release‐activated Ca^2+^ modulator 1; *RYR1*: ryanodine receptor 1; *S100A1*: S100 Ca^2+^ binding protein A1; *SCN4A*: Na^+^ voltage‐gated channel alpha subunit 4; *SLC8A1*: solute carrier family 8 member A1; *SLN*: sarcolipin; *STIM1*: stromal interaction molecule 1; *TRDN*: triadin. mRNA levels were measured by qPCR. Values are expressed as fold‐change (FC) of DI5‐to‐Pre values. Data were analysed by a two tailed paired *t*‐test except for *ATP1A2*, *ATP2A1*, *CALU*, *CASQ1*, *CLCN1*, *S100A1*, and *SLC8A1* mRNA levels, which were analysed by a two tailed Wilcoxon test.
