# Supplementary material for: Molecular determinants of skeletal muscle force loss in response to 5 days of dry immersion in human
Source: J Cachexia Sarcopenia Muscle. 2024 Oct 25;15(6):2323–37. doi: 10.1002/jcsm.13559 (PMC11634509; doi:10.1002/jcsm.13559)
Supplement: Supplementary file 1 — Table S1. Primer sequences used for qPCR analyses. 18S: 18S ribosomal RNA; AQP4: aquaporin 4; ATG5: autophagy related 5; ATP1A1 and ATP1A2: ATPase Na+/K+ transporting subunit alpha 1 and 2; ATP2A1 and 2: ATPase sarcoplasmic/endoplasmic reticulum Ca2+ transporting 1 and 2 (SERCA1 and SERCA2); ATP2B1: ATPase plasma membrane Ca2+ Transporting 1 (PMCA1); B2M: beta‐2‐microglobulin; BNIP3: BCL2 interacting protein 3; CACNA1S: Ca2+ voltage‐gated channel subunit alpha 1 S; CACNA2D1: Ca2+ voltage‐gated channel auxiliary subunit alpha 2 delta 1; CALM1: calmodulin 1; CALU: calumenin; CASQ1: calsequestrin 1; CHRNA1: cholinergic receptor nicotinic alpha 1 subunit; CLCN1: chloride voltage‐gated channel 1; CTSB: cathepsin B; CTSL: cathepsin L; DDIT4: DNA damage inducible transcript 4; FBXO30: F‐box protein 30; FBXO32: F‐box protein 32 (MAFBX); GABARAPL1: GABA type A receptor associated protein like 1; JPH2: junctophilin 2; JSRP1: junctional sarcoplasmic reticulum protein 1; MYH1, MYH2 and MYH7: myosin heavy chain 1, 2 and 7 (MHC2A, MHC2X and MHC1, respectively); MYL2 and MYL11: myosin light chain 2 and 11; ORAI1: ORAI Ca2+ release‐activated Ca2+ modulator 1; PSMA7: 20S proteasome subunit alpha 7; PSMB4 and PSMB7: 20S proteasome subunit beta 4 and beta 7; RYR1: ryanodine receptor 1; S100A1: S100 Ca2+ binding protein A1; SCN4A: Na+ voltage‐gated channel alpha subunit 4; SLC8A1: solute carrier family 8 member A1; SLN: sarcolipin; STIM1: stromal interaction molecule 1; TNNC1 and TNNC2: troponin C 1 and 2; TNNI1 and TNNI2: Troponin I 1 and 2; TNNT1 and TNNT 3: troponin T 1 and 3; TPM1 and TPM2: tropomyosin 1 and 2; TRDN: triadin; TRIM63: tripartite motif containing 63 (MURF1). [file JCSM-15-2323-s003.docx]

**Supplementary Table 1.** Primer sequences used for qPCR analyses. *18S*: 18S ribosomal RNA; *AQP4*: aquaporin 4; *ATG5*: autophagy related 5;  *ATP1A1* and *ATP1A2*: ATPase Na^+^/K^+^ transporting subunit alpha 1 and 2; *ATP2A1* and *2*: ATPase sarcoplasmic/endoplasmic reticulum Ca^2+^ transporting 1 and 2 (SERCA1 and SERCA2); *ATP2B1*: ATPase plasma membrane Ca^2+^ Transporting 1 (PMCA1); *B2M*: beta-2-microglobulin; *BNIP3*: BCL2 interacting protein 3; *CACNA1S*: Ca^2+^ voltage-gated channel subunit alpha 1 S; *CACNA2D1*: Ca^2+^ voltage-gated channel auxiliary subunit alpha 2 delta 1; *CALM1*: calmodulin 1; *CALU*: calumenin; *CASQ1*: calsequestrin 1; *CHRNA1*: cholinergic receptor nicotinic alpha 1 subunit; *CLCN1*: chloride voltage-gated channel 1; *CTSB*: cathepsin B; *CTSL*: cathepsin L; *DDIT4*: DNA damage inducible transcript 4; *FBXO30*: F-box protein 30; *FBXO32*: F-box protein 32 (MAFBX); *GABARAPL1*: GABA type A receptor associated protein like 1; *JPH2*: junctophilin 2; *JSRP1*: junctional sarcoplasmic reticulum protein 1; *MYH1*, *MYH2* and *MYH7*: myosin heavy chain 1, 2 and 7 (MHC2A, MHC2X and MHC1, respectively); *MYL2* and *MYL11*: myosin light chain 2 and 11; *ORAI1*: ORAI Ca^2+^ release-activated Ca^2+^ modulator 1; *PSMA7:* 20S proteasome subunit alpha 7; *PSMB4* and *PSMB7*: 20S proteasome subunit beta 4 and beta 7; *RYR1* : ryanodine receptor 1; *S100A1*: S100 Ca^2+^ binding protein A1; *SCN4A*: Na^+^ voltage-gated channel alpha subunit 4; *SLC8A1*: solute carrier family 8 member A1; *SLN*: sarcolipin; *STIM1*: stromal interaction molecule 1; *TNNC1* and *TNNC2*: troponin C 1 and 2; *TNNI1* and *TNNI2*: Troponin I 1 and 2; *TNNT1* and *TNNT 3*: troponin T 1 and 3; *TPM1* and *TPM2*: tropomyosin 1 and 2; *TRDN*: triadin; *TRIM63*: tripartite motif containing 63 (MURF1).

| Gene | Forward | Reverse | NCBI RefSeq |
| --- | --- | --- | --- |
| *18S* | AGTCCCTGCCCTTTGTACACA | GATCCGAGGGCCTCACTAAAC | NR_145819.1 |
| *AQP4* | CATGGAAATCTTACCGCTGGT | TCAGTCCGTTTGGAATCACAG | NM_001317387.2 |
| *ATG5* | GCAAGCCAGACAGGAAAAAG | GACCTTCAGTGGTCCGGTAA | NM_001286111.1 |
| *ATP1A1* | ACAGACTTGAGCCGGGGATTA | TCCATTCAGGAGTAGTGGGAG | NM_000701.8 |
| *ATP1A2* | GCCGCAAATACCAAGTGGAC | AAGCTGACGGCAGAACTTGA | NM_000704.4 |
| *ATP2A1* | GGTCAGAGCTTTGTGGAGGG | CCTGTCTGCCTGGGTTTCTT | NM_004320.6 |
| *ATP2A2* | GCTGAAAATCTCCTTGCCCG | CCAGTATTGCAGGTTCCAGGT | NM_001681.4 |
| *ATP2B1* | GAGCTGCGGGCTCTCATG | TGGTGCAAATTCCATAGACATCTC | NM_001366530.1 |
| *B2M* | GAGGCTATCCAGCGTACTCCA | CGGCAGGCATACTCATCTTTT | NM_004048.2 |
| *BNIP3* | GCTGTTAGATCCTGGGGTGG | GTCTGACATGCTTATTGATCCCA | NM_004052.3 |
| *CACNA1S* | GAGACCCCTGGAATGTGTTTG | CCTCCACCCAGGCAATACAGT | NM_000069.3 |
| *CACNA2D1* | CCGTCACTATCAAATCATGGGTG | TGCTTGAACTTTCTCCGCTTC | NM_000722.4 |
| *CALM1* | TTGACTTCCCCGAATTTTTGACT | GGAATGCCTCACGGATTTCTT | NM_006888.6 |
| *CALU* | TGGGATGAGTACAGAAACGTGA | CCTTGGTGGCAATGAGGTCT | NM_001130674.3 |
| *CASQ1* | TGCGGATAGCGTATGGATGG | CAGTGGGGCTGAAAGATGGT | NM_001231.5 |
| *CHRNA1* | GCAGAGACCATGAAGTCAGACCAGGAG | TTCATGCTTGTTTGCATCATCGG | NM_000079.4 |
| *CLCN1* | GATCCTTTGACCCCCTCCCA | CATGACCTCGCCACATTCAG | NM_000083.3 |
| *CTSB* | TCCGGCAACGCCAACC | GATCCTAGATCCACCCAGCG | NM_001908.5 |
| *CTSL* | GTCTACCCCGAACTCTGCTG | TTAAAACCTGTGTCCACCCG | NM_001912.5 |
| *DDIT4* | TGGGCAAAGAACTACTGCG | AGAGTTGGCGGAGCTAAACAG | NM_019058.3 |
| *FBXO30* | TGGCCCGAAATAAAGTTGCTG | CCGGTCTGCATAACTAACTGG | NM_032145.5 |
| *FBXO32* | TTCAAACTTGTCCGATGTTACCC | CCAGGAAAGGATGTGACAGTGT | NM_148177.2 |
| *GABARAPL1* | CCGGAAGAGAATCCACCTGAG | CACTCTCATCACTGTAGGCCA | NM_031412.4 |
| *JPH2* | GTATGAGGGCACCTGGAACAA | TGGTGAACTGGCCTTGGT | NM_020433.5 |
| *JSRP1* | TGTCGCTCAACAAGTGCCTG | GCCTGGGCCTCGAACTTAG | NM_144616.4 |
| *MYH1* | ATCTCTACGCCAGGGTCCTTA | GAGCAGCCTCCCCAAAAATG | NM_005963.4 |
| *MYH2* | CTGAGGGAGGAGCGACTCT | CTCGGGCTTATACACAGGCA | NM_017534.6 |
| *MYH7* | GTAGACACACTTGAGTAGCCC | GGTCAAAAGGCCTGGTCTG | NM_000257.4 |
| *MYL11* | CTCCTTGCTTCTTTCCAGCC | CCGCCCTCTACTGTCCTTCT | NM_013292.5 |
| *MYL2* | AATTCTTCTCGGGAGGCAGTG | GAAGCCATCCCTGTTCTGGT | NM_000432.4 |
| *ORAI1* | GGACGCTGACCACGACTAC | GGGACTCCTTGACCGAGTT | NM_032790.3 |
| *PSMA7* | ACCGCCGATGCAAGGATAG | CTGCGTATAACGCTGCTTCAG | NM_002792.4 |
| *PSMB4* | GGAGAAGCAGCCAGTGCTAA | CTTTTTCGGTGACAGTGGCG | NM_002796.3 |
| *PSMB7* | CTGTGTCGGTGTATGCTCCA | CGATGGTCGTGCCAGTTTTC | NM_002799.4 |
| *RYR1* | ACTGGTCCAAGTCCCACAAC | CCGCCTTAGCCATTTTGCTT | NM_000540.3 |
| *S100A1* | ACATTTGCAACCTTGGCCATC | ACACGTTGATGAGGGTCTCC | NM_001024210.2 |
| *SCN4A* | TCGGTGAAAAAGCTGTCGGA | AGTCCTACCAGCGCAAAGAC | NM_000334.4 |
| *SLC8A1* | ACAACATGCGGCGATTAAGTC | GCTCTAGCAATTTTGTCCCCA | NM_001351493.2 |
| *SLN* | GCGAGGAGAAACCTGCCATA | TTTCTGAGGGCACACCAAGG | NM_003063.3 |
| *STIM1* | TCACAGTGAGAAGGCGACAG | GTGGATGTTACGGACTGCCT | NM_001382567.1 |
| *TNNC1* | ATGGTTCGGTGCATGAAGGA | CCAGGTCGATGTAGCCATCA | NM_003280.3 |
| *TNNC2* | AGGCGACCTGCAACAGAGG | CACCACCATCAGCATCAAACAT | NM_003279.3 |
| *TNNI1* | GAGGTGTTCCAACCTGGGAG | ACGCATACACACCATGCTCA | NM_003281.4 |
| *TNNI2* | GCCCTGCTGCCCAGATT | CCTGTTCCGCTTCTCCTCAT | NM_003282.4 |
| *TNNT1* | CACAGCATCTCAGACACAGC | CGGGGCGGCTTGGTTT | NM_001126133.3 |
| *TNNT3* | CTCACACTCGACCCGCAGAG | CCCCACGAGCCAGTGTTGT | NM_001297646.2 |
| *TPM1* | TGCTGAAGCTCGACAAGGAG | GACACCCGCAGCAACTTCTC | NM_001365778.1 |
| *TPM2* | AACTCAACAACCTGTGAGGGC | AGGGCCTTGAGAGGCTAGTAAC | NM_213674.1 |
| *TRDN* | TCACAGAAGACATAGTGACGACG | TGGCAATAGAGCTTGCTGAAA | NM_001256021.2 |
| *TRIM63* | ACCTGCTGGTGGAAAACATC | AGGAGCAAGTAGGCACCTCA | NM_032588.3 |
