# Supplementary material for: Molecular determinants of skeletal muscle force loss in response to 5 days of dry immersion in human
Source: J Cachexia Sarcopenia Muscle. 2024 Oct 25;15(6):2323–37. doi: 10.1002/jcsm.13559 (PMC11634509; doi:10.1002/jcsm.13559)
Supplement: Supplementary file 2 — Table S2. List of antibodies. 4E‐BP1Thr37/46: phosphorylated eIF4E‐binding protein 1; AQP4: aquaporin 4; ATP1A2: ATPase Na+/K+ transporting subunit alpha 2; NCX: Na+/Ca2+ exchanger; RPS6: Ribosomal protein S6; RPS6Ser235/236 phosphorylated ribosomal protein S6; TnCs: troponin C slow isoform; TnCf: troponin C fast isoform; TnIs: troponin I slow isoform; TnIf: troponin I fast isoform; TnTs: troponin T slow isoform; TnTf: troponin T fast isoform; TRPC1: transient receptor potential cation channel subfamily C member 1. [file JCSM-15-2323-s002.docx]

**Supplementary Table 2.** List of antibodies. 4E-BP1^Thr37/46^: phosphorylated eIF4E-binding protein 1; AQP4: aquaporin 4; ATP1A2: ATPase Na^+^/K^+^ transporting subunit alpha 2; NCX: Na^+^/Ca^2+^ exchanger; RPS6: Ribosomal protein S6; RPS6^Ser235/236^ phosphorylated ribosomal protein S6; TnCs: troponin C slow isoform; TnCf: troponin C fast isoform; TnIs: troponin I slow isoform; TnIf: troponin I fast isoform; TnTs: troponin T slow isoform; TnTf: troponin T fast isoform; TRPC1: transient receptor potential cation channel subfamily C member 1.

| Antibody | Catalog number | Manufacturer/donator | Antibody dilution |
| --- | --- | --- | --- |
| 4E-BP1^Thr37/46^ | 9459S | Cell Signaling Technology | 1:1,000 |
| StarBright^TM^ blue 700 | #12005870 | Bio-Rad | 1:2,000 |
| StarBright^TM^ blue 520 | #12004158 | Bio-Rad | 1:2,000 |
| Anti-mouse HRP | #7076 | Cell Signaling Technology | 1:5,000-1:10,000 |
| AQP4 | SC-32739 | Santa Cruz Biotechnology | 1:1,000 |
| ATP1A2 | A3747 | ABclonal | 1:1,000 |
| NCX | MA3-926 | Invitrogen | 1:1,000 |
| RPS6 | 2217S | Cell Signaling Technology | 1:1,000 |
| RPS6^Ser235/236^ | 4856S | Cell Signaling Technology | 1:1,000 |
| TnC slow and fast | SC-48347 | Santa Cruz Biotechnology | 1:5,000 |
| TnI slow and fast | - | Pr. JP Jin, Wayne State University, USA | 1:5,000 |
| TnTf | - | Pr. JP Jin, Wayne State University, USA | 1:5,000 |
| TnTs | - | Pr. JP Jin, Wayne State University, USA | 1:5,000 |
| TRPC1 | SC-133076 | Santa Cruz Biotechnology | 1:1,000 |
