# Supplementary material for: Molecular determinants of skeletal muscle force loss in response to 5 days of dry immersion in human
Source: J Cachexia Sarcopenia Muscle. 2024 Oct 25;15(6):2323–37. doi: 10.1002/jcsm.13559 (PMC11634509; doi:10.1002/jcsm.13559)
Supplement: Supplementary file 3 — Table S3 Effects of 5 days of DI on body weight, maximal heart rate, maximal oxygen consumption (VO2max) and maximal aerobic power. DI5: 5 days of dry immersion. Values are means ± SD and were analysed by two tailed paired t‐test. [file JCSM-15-2323-s001.docx]

**Supplementary Table 3** Effects of 5 days of DI on body weight, maximal heart rate, maximal oxygen consumption (VO_2_max) and maximal aerobic power. DI5: 5 days of dry immersion. Values are means ± SD and were analyzed by two tailed paired *t*-test.

|  | Pre | DI5 | % | *P* value |
| --- | --- | --- | --- | --- |
| Body weight (kg) | 74.1 ± 8.0 | 72.4 ± 7.9 | -2.3 | < 0.0001 |
| Max. heart rate (bpm) | 181.6 ± 8.9 | 186.2 ± 8.3 | 2.5 | 0.0209 |
| VO_2_max (mL.min^-1^.kg^-1^) | 47.4 ± 6.4 | 43.3 ± 5.2 | -8.6 | < 0.0001 |
| Max. aerobic power (W) | 272.6 ± 31.0 | 246.9 ± 24.5 | -9.4 | 0.0003 |
