# Supplementary material for: Molecular determinants of skeletal muscle force loss in response to 5 days of dry immersion in human
Source: J Cachexia Sarcopenia Muscle. 2024 Oct 25;15(6):2323–37. doi: 10.1002/jcsm.13559 (PMC11634509; doi:10.1002/jcsm.13559)
Supplement: Supplementary file 4 — Table S4 Fold‐change in mRNA level. AQP4: aquaporin 4; ATP1A1 and ATP1A2: ATPase Na+/K+ transporting subunit alpha 1 and 2; ATP2A1 and 2: ATPase sarcoplasmic/endoplasmic reticulum Ca2+ transporting 1 and 2 (SERCA1 and SERCA2); ATP2B1: ATPase plasma membrane Ca2+ Transporting 1 (PMCA1); CACNA1S: Ca2+ voltage‐gated channel subunit alpha 1 S; CACNA2D1: Ca2+ voltage‐gated channel auxiliary subunit alpha 2 delta 1; CALM1: calmodulin 1; CALU: calumenin; CASQ1: calsequestrin 1; CHRNA1: cholinergic receptor nicotinic alpha 1 subunit; CLCN1: chloride voltage‐gated channel 1; JPH2: junctophilin 2; JSRP1: junctional sarcoplasmic reticulum protein 1; ORAI1: ORAI Ca2+ release‐activated Ca2+ modulator 1; RYR1: ryanodine receptor 1; S100A1: S100 Ca2+ binding protein A1; SCN4A: Na+ voltage‐gated channel alpha subunit 4; SLC8A1: solute carrier family 8 member A1; SLN: sarcolipin; STIM1: stromal interaction molecule 1; TRDN: triadin. mRNA levels were measured by qPCR. Values are expressed as fold‐change (FC) of DI5‐to‐Pre values. Data were analysed by a two tailed paired t‐test except for ATP1A2, ATP2A1, CALU, CASQ1, CLCN1, S100A1, and SLC8A1 mRNA levels, which were analysed by a two tailed Wilcoxon test. [file JCSM-15-2323-s004.docx]

**Supplementary Table 4** Fold-change in mRNA level. *AQP4*: aquaporin 4; *ATP1A1* and *ATP1A2*: ATPase Na^+^/K^+^ transporting subunit alpha 1 and 2; *ATP2A1* and *2*: ATPase sarcoplasmic/endoplasmic reticulum Ca^2+^ transporting 1 and 2 (SERCA1 and SERCA2); *ATP2B1*: ATPase plasma membrane Ca^2+^ Transporting 1 (PMCA1); *CACNA1S*: Ca^2+^ voltage-gated channel subunit alpha 1 S; *CACNA2D1*: Ca^2+^ voltage-gated channel auxiliary subunit alpha 2 delta 1; *CALM1*: calmodulin 1; *CALU*: calumenin; *CASQ1*: calsequestrin 1; *CHRNA1*: cholinergic receptor nicotinic alpha 1 subunit; *CLCN1*: chloride voltage-gated channel 1; *JPH2*: junctophilin 2; *JSRP1*: junctional sarcoplasmic reticulum protein 1; *ORAI1*: ORAI Ca^2+^ release-activated Ca^2+^ modulator 1; *RYR1*: ryanodine receptor 1; *S100A1*: S100 Ca^2+^ binding protein A1; *SCN4A*: Na^+^ voltage-gated channel alpha subunit 4; *SLC8A1*: solute carrier family 8 member A1; *SLN*: sarcolipin; *STIM1*: stromal interaction molecule 1; *TRDN*: triadin. mRNA levels were measured by qPCR. Values are expressed as fold-change (FC) of DI5-to-Pre values. Data were analyzed by a two tailed paired *t*-test except for *ATP1A2*, *ATP2A1*, *CALU*, *CASQ1*, *CLCN1*, *S100A1*, and *SLC8A1* mRNA levels, which were analyzed by a two tailed Wilcoxon test.

| Variable | FC | *P* value |
| --- | --- | --- |
| *AQP4* | 0.774 | 0,060 |
| *ATP1A1* | 0.853 | 0,222 |
| *ATP1A2* | 0.888 | 0,562 |
| *ATP2A1* | 0.950 | 0,926 |
| *ATP2A2* | 0.675 | 0,002 |
| *ATP2B1* | 0.809 | 0,016 |
| *CACNA1S* | 0.900 | 0,095 |
| *CACNA2D1* | 0.765 | 0,007 |
| *CALM1* | 1.053 | 0,457 |
| *CALU* | 0.706 | 0,025 |
| *CASQ1* | 0.851 | 0,025 |
| *CHRNA1* | 1.751 | 0,014 |
| *CLCN1* | 0.922 | 0,720 |
| *JPH2* | 0.758 | 0,008 |
| *JSRP1* | 1.250 | 0,002 |
| *ORAI1* | 0,638 | 0,001 |
| *RYR1* | 1,034 | 0,626 |
| *S100A1* | 1,272 | 0,009 |
| *SCN4A* | 1,009 | 0,890 |
| *SLC8A1* | 1,000 | 0,940 |
| *SLN* | 1,198 | 0,113 |
| *STIM1* | 1,017 | 0,814 |
| *TRDN* | 0,844 | 0,053 |
